# Assessing Environmental Risks of Local Contamination of Garden Urban Soils with Heavy Metals Using Ecotoxicological Tests

**DOI:** 10.3390/toxics12120873

**Published:** 2024-11-30

**Authors:** Dariusz Gruszka, Iwona Gruss, Katarzyna Szopka

**Affiliations:** 1 Institute of Soil Science, Plant Nutrition and Environmental Protection, Wroclaw University of Environmental and Life Sciences, 50-375 Wroclaw, Poland; katarzyna.szopka@upwr.edu.pl; 2 Department of Plant Protection, Wroclaw University of Environmental and Life Sciences, 50-375 Wroclaw, Poland; iwona.gruss@upwr.edu.pl

**Keywords:** urban gardens, soil contaminated with trace materials, Ostracodtoxkit, ecotoxicology, urban areas

## Abstract

Heavy metal soil contamination in urban areas poses a significant environmental hazard, particularly in regions with historical or ongoing industrial activities. These areas are often polluted with metals such as Pb, Cu, Cd, and Zn, which can be absorbed by plants and pose risks to both ecosystems and human health. This study investigates soil contamination in urban gardens in Wroclaw, Poland, where elevated levels of trace elements were detected. Standard soil analyses, including macroelement content, granulometry, and trace element concentrations, were performed alongside an ecotoxicological evaluation using an Ostracodtoxkit test. The test evaluates the impact of contaminants on organism growth. An uncontaminated urban garden soil served as a reference. This study revealed that Zn, Cu, Pb, and Cd concentrations in soils exceeded limits permitted by Polish regulations in several soil samples. Despite the high concentrations of total metals, the bioavailable forms of these metals (measured by extraction of 1 M NH_4_NO_3_ extraction) were significantly lower, highlighting that the total metal content may not fully reflect the environmental risk. Pb was identified as the primary contributor to growth inhibition of test organisms, showing a particularly strong correlation with ecotoxicity. These findings underscore the importance of using ecotoxicological tests to evaluate soil contamination risks.

## 1. Introduction

The contamination of soils with heavy metals in urban areas is a serious hazard [1,2,3]. In particular, areas with the current or historical development of heavy industry are the most polluted [4,5,6]. The rapid development of urban areas as housing, transport, and commercial and service facilities contributes to the contamination of the environment with heavy metals [7]. There are many examples in the world of environmental hazards related to exposure to heavy metals in urban areas. For example, in Toronto (Canada), Mexican cities, and India, it was found that children are exposed to Pb in soil and water, originating from past industrial activities [3,8,9,10,11,12], respectively. In northwest China, exceeded concentrations of Cu and Zn have been found in traffic-source soils, while exceeded concentrations of Pb, Hg, and As have been sourced from industrial activities [6].

The main concern is urban gardening, which gained significant relevance due to its economic, social, and cultural benefits [13]. The contamination of these soils with heavy metals and their use for growing fruits and vegetables could be a threat to people’s health [14,15,16,17,18,19,20,21]. Chronic symptoms of lead Pb to human disease include damage to the kidney, brain, reproductive organs, and CNS/PNS [22,23]. Long-term exposure to cadmium leads to cancer and toxicity symptoms in organ systems [24]. The human body requires certain metal cations for proper functioning. These can be categorised into essential metals, which play a well-defined role in metabolic processes, and nonessential metals, which have no beneficial function. Essential metals, such as copper (Cu), sodium (Na), magnesium (Mg), iron (Fe), potassium (K), and calcium (Ca), are needed in the diet, and their deficiency can cause imbalances in metabolism and deficiency diseases. Nonessential metals, such as mercury (Hg), gold (Au), nickel (Ni), chromium (Cr), and lead (Pb), are toxic and pose health risks. These harmful metals can accumulate in vegetables grown in contaminated soil, and when consumed, they accumulate in the human body [25,26].

Heavy metal contamination also causes environmental threats. Exceeded concentrations of Pb, Cu, Zn, and Cd negatively affect plant growth [14]. Lead compounds have a negative impact on microbiological activity and plant growth. It disrupts photosynthesis and water absorption by root systems and limits the availability of some biogenic elements for plants [23,27]. Cadmium contributes to the disruption of homeostasis, the intensification of oxidative stress in plant cells, and the disruption of their membrane integrity [28,29]. However, plants have developed complex mechanisms to overcome these biotic and abiotic stresses during evolution, including, for example, hyperaccumulation or tolerance [30]. Plants must evolve rapidly to adapt to environmental challenges, such as heavy-metal-rich soils, which may be naturally enriched or anthropogenically contaminated. Some species thrive under these conditions through hyper-tolerance mechanisms. One strategy involves excluding toxic metals, minimising absorption and root-to-shoot translocation [31]. Alternatively, hyperaccumulators can store two to four times more metals in their shoots than non-accumulating plants in the same environment [32,33]. These plants tolerate high levels of metals without toxicity symptoms, accumulating >10 mg/g Mn, >3 mg/g Zn, >1 mg/g As, Cr, Ni, or Pb; >0.3 mg/g Co or Cu; and >0.1 mg/g Cd, Se, or Tl in their shoots. Hyperaccumulators remove metals from roots by storing them in aerial tissues [34].

Certain plant species tend to accumulate heavy metals in their tissues, causing a serious threat to humans from their consumption [10]. Vegetables growing near the current or historical industry [34,35] or highways [9] were more contaminated compared to those originating from low-contaminated sites.

Ecotoxicological tests are valuable tools for assessing the impact of soil pollution on organisms that inhabit or interact with contaminated soils. They provide a comprehensive evaluation of soil toxicity and its broader ecological implications [23,36]. An effective assessment of soil contamination should include a combination of chemical and ecotoxicological indices [37,38]. Toxicological bioassays provide much more reliable information on the effects exerted by environmental pollution on the biota than soil chemistry alone, as they are based on reactions of living organisms to complex sets of factors that can act synergistically or antagonistically [39]. The ecotoxicological examination of environmental samples should therefore be an important complement to chemical analysis in a comprehensive assessment of environmental risk [40]. Numerous assays, which use various groups of biota, representative of different levels of the food chain, have been adopted as ISO or OECD norms [41,42,43].

The useful tool for assessing the toxicity of bioavailable forms of the trace elements is the Ostracodtoxkit (*Heterocypris incongruens*). *H. incongruens* was a more sensitive indicator of soil contamination with trace elements than test plants [40]. In another study, crustacean bioassays (including *H. incongruens*) were more sensitive than the bacterial test [41]. The toxicity of trace elements on organisms depends on the forms of their occurrence in soils and soil properties, as well as factors affecting the solubility of trace elements and their concentration in soil solutions [44,45]. Trace element concentrations in soils are determined primarily by their levels in the parent material from which the soils formed. However, anthropogenic activities (responsible for the release of trace elements in ecosystems) often modify the natural level, biochemical balance, and geochemical cycling of these metals in the environment [46]. Heavy metals can be involved in a series of complex chemical and biological interactions. The most important factors that affect the mobility of trace elements are pH, sorbent nature, presence and concentration of organic and inorganic ligands, root exudates, and nutrients. Furthermore, redox reactions, both biotic and abiotic, are of great importance in controlling the oxidation state and, thus, the mobility and the toxicity of many elements, such as Cr, Se, Co, Pb, As, Ni, and Cu [38]. The bioavailability of elements in the soil reflects the extent to which an element present in a potential source of contamination (in this case, in the soil) can enter into a plant or other living organisms or be absorbed by them [47]. Bioavailability is the proportion of total metals available for incorporation into the biota (bioaccumulation). Total metal concentrations do not necessarily correspond to metal bioavailability. For this reason, it is important to assess the bioavailability of elements by soil organisms, for which we use ecotoxicological tests [47,48].

The objective of this investigation was to evaluate the environmental risk of trace element contamination in garden soils using inhibition of ostracod growth as the toxicity response. The following hypotheses were tested:

1. Ecotoxicological tests using *Heterocypris incongruens* offer a more sensitive evaluation of soil contamination compared to traditional chemical analyses.
2. The toxicity of trace elements to organisms depends on their chemical forms in the soil and environmental factors that affect their solubility and concentration in soil solutions. These factors include soil processes such as sorption, mobility, and bioavailability, influenced by geochemical, climatic, and biological conditions.
3. Local contamination significantly increases environmental risk, as demonstrated by the results of ecotoxicological assessments.

## 2. Materials and Methods

### 2.1. Soils

**Figure 1:**
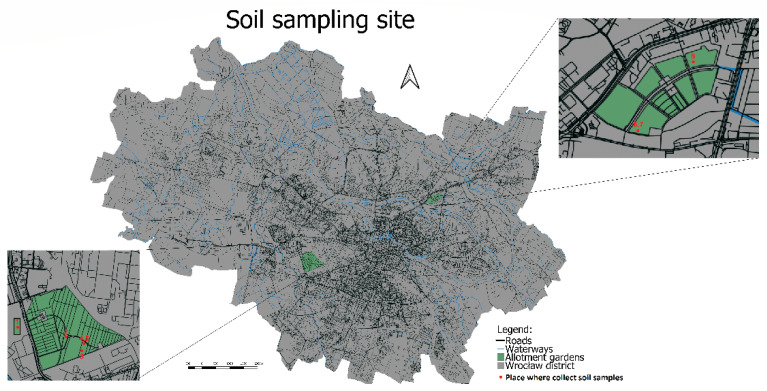
Location of the study area.

The soil material was collected from three urban gardens located in Wroclaw (Poland), in total from 8 sampling points (1–8) (Figure 1). Detailed data on the location and soil conditions are given in Figure 1 and Table 1. The gardens were selected based on the high risk of local heavy metal contamination related to historical or current industry, high road traffic, and the flood that occurred in 1997. In flood-affected areas, sediment sorting and mixing of pollutants with grains from diverse sources within a catchment pose significant challenges. This process can cause metal concentrations to vary by one to two orders of magnitude in just a few centimetres. During floods, pollutants formerly temporarily stored in the channel are quickly entrained and transferred to the floodplain [49]. Floods can also trigger primary pollution when extreme precipitation causes settling ponds or washes of stockpiles [48]. No contamination was found in the soil at point 1; this soil is close to the arterial road, and points 1–4 are close to industrial sites, such as Hutmen, which is used to process copper materials. Plots 6–7 are close to an establishment that in the past dealt with car repairs and paint services. Allotment holders reported that during the 1997 flood, a large amount of car paint was spread over the southern part of the allotments, hence possible contamination. Point 8 is located close to a residential area.

The soil samples were taken from levels 0–25 cm, and the average weight of one sample was 2 kg. Samples were taken from areas intended for vegetable cultivation, three from each plot (garden) in July 2022. The samples were dried and sieved through a 2 mm sieve to separate the skeletal fraction from the earth fraction. The earth fraction is the sum of fractions with a diameter equal to or less than <2 mm, while the skeletal fraction is the fraction above >2 mm. The soil texture was determined using a combined sieve and hydrometer method and chemical analyses were performed with commonly used methods [50]. The soil pH was measured in a suspension in 1 M KCl (1:2.5; *v*/*v*). Organic carbon (Corg) and carbonates were determined using a CS-MAT 5500 analyser (Strohlein Instruments, which is based in Kaarst, Germany).

Total trace metal concentrations were determined after soil digestion with aqua regia in a microwave system. The concentrations of Cd, Cu, Pb, and Zn in the digests were determined by ICP-AES (iCAP 7400, Thermo Fisher Scientific, Waltham, MA, USA). All analyses were performed in triplicates. The quality of the determinations has been monitored using soil reference materials (sample ID: SRM 2709, SRM 2711, RTH 912, RTH 953), with the certified total concentration (‘aqua regia extractable’) of trace elements being analysed. Potentially soluble forms were extracted in dilute nitric acid according to the ISO standard [51] and in 1 M ammonium nitrate according to the ISO standard [52]. The concentrations of other possible pollutants were not measured.

**Table 1:** The basic parameters of studies soils (1–8).

NO	GR. GROUP	FLA. FRA	FRA	pH H_2_O	pH KCl	C [g/kg]	N [g/kg]	C/N	HYD
1	SCL	24	4.4	6.91	6.71	3.64	0.25	14.66	0.1
2	SCL	26	-	7.25	6.87	4.43	0.25	17.57	0.54
3	SL	15	-	7.48	6.80	3.88	0.31	12.53	0.35
4	SCL	21	8.4	7.94	7.16	4.10	0.22	18.37	0
5	SCL	23	6.7	8.00	7.14	7.45	0.40	18.49	0
6	LS	16	4.4	7.04	6.92	6.43	0.34	18.86	0.39
7	LS	17	5.6	6.9	6.87	7.64	0.37	20.68	0.2
8	SCL	20	8.2	7.78	7.58	3.64	0.25	14.66	0

Note: Explanation of the abbreviations used in Table 2: NO—No. of sampling point, GR. GROUP—Granulometric Group (USDA clasyfication [53]), FLA. FRA—Flatable fraction (<0.02 [mm] [%]), FRA—Fraction (>2 [mm] [%]), pH H_2_O—pH H_2_O Destilated—mean, pH KCl—pH KCl—mean, C [g/kg]—C [g/kg]—mean, N [g/kg]—N [g/kg]—mean, HYD—Hydrolitic acidity.

The physical and chemical characteristics of the soils from sampling points 1–8 were similar (Table 1). Soils were classified as SCL (sandy clay loam—sampling points 1, 2, 4, 5, 8), SL (sandy loam—sampling point 3), and LS (loamy sand—sampling points 6, 7) (according to USDA classification) [54]. The pH in KCl ranged from 6.71 to 7.58, which corresponds to the appropriate level for plant development and does not significantly contribute to the mobility of heavy metals. The N content [g/kg] ranged from 0.22 to 0.40. The C content [g/kg] ranged from 3.64 to 7.64, which is at the appropriate level considering urban gardening, and the difference is the result of the soil additives that improve plant growth (for example, manure or compost).

### 2.2. The Toxicity Tests

To increase the bioavailability of heavy metals in the tested soils, the soils were previously irrigated for 1 week to 70% of the water capacity. The toxicity of eight test soils (1–8, Table 1) was tested, and a reference sediment marked K was used as an additional control. The reference sediment is the standard control recommended in the Ostracod test.

A standardised Ostracod test kit was used for ecotoxicological tests (ISO Standard 14371) [54]. The test organisms were *Heterocypris incongruens*, a crustacean of the phylum Ostracoda. One repetition was a test well filled with test soil (1000 µL) flooded with 4 mL of standard medium with food (algae). There were ten test organisms per replicate. Each variant of the experiment (1–8, K) was repeated six times.

The test organisms are juvenile ostracods obtained by activation of cysts for an incubation time of 48 h at T [25 °C], 3000 LUX illumination. The average size of the juveniles on the day the test began was 160 micrometres. The duration of the test is 6 days from the moment the ostracods are placed in the test wells.

After 6 days, the ostracods of each test well were immobilised in Lugol solution and measured under a microscope (Zeiss Stemi 508 is manufactured by Carl Zeiss AG, headquartered in Oberkochen, Germany) with a microscope camera (Axiocam ERc 5s is manufactured by Carl Zeiss AG, headquartered in Oberkochen, Germany) using object measurement software (Zen Core 3.5).

### 2.3. Data Analysis

The normality of the data was confirmed using a Shapiro–Wilk test. The body size of the ostracods was compared using analysis of variance (ANOVA). Differences between individual combinations were checked using a Tukey’s post hoc test. The analyses were performed in SAS University Edition. The inhibition of growth rate of the test organisms was calculated using the following formula:

$$Inhibition\;of\;growth=100-((\frac{Average\;growth\;rate}{Growth\;rate\;in\;reference\;sediment})\cdot 100)$$

where

Average growth rate—the average of the growth rates of all organisms in the sample (μm);

Growth rate in the reference sediment—constant = 203.44.

Pearson’s correlation was evaluated to investigate the relationship between growth inhibition effects and individual heavy metals in soils, considering both total and bioavailable forms. A species-sensitivity distribution (SSD) approach was employed to assess the sensitivity of test species to contaminated soils. This involved calculating the potentially affected fraction (PAF) of individuals—representing the proportion of individuals experiencing growth inhibition—based on contaminant concentrations in soil, following the methodology of [55]. Various fitting models were tested to determine the best relationship between contaminant concentration and PAF, with polynomial modelling providing the highest R^2^ value. All analyses were performed with the Origin Pro software 10.2.

## 3. Results

### 3.1. The Concentrations of Trace Metals and Their Available Forms Content in the Studied Examined Soils

Soil contamination with Zn, Cu, Pb, and Cd was compared with the permissible limits set by Polish legislation [54] (Table 2), with additional limits for Cu, Zn, Pb, and Cd in agricultural soils in various countries presented in Table S1 [56,57,58,59]. While most European countries have legislation specifying permissible element concentrations in soils, the methods to determine these limits vary greatly (Table S1).

According to the Polish permissible limits, Zn concentrations exceeded the permissible levels at sampling points 2, 3, 4, 5, 6, 7, and 8, with values ranging from 1059 to 3465 mg/kg (dry mass). The permissible Cu content was exceeded at sampling point 2 with a concentration of 322 mg/kg (dry mass). An elevated Pb content was observed in sample 6, 7, 8, with levels ranging from 329 to 402 mg/kg (dry mass). The permissible Cd content was exceeded at sampling points 4, and 5, with a concentration of 6 mg/kg (dry mass) (Table 2). Compared to the permissible limits for other countries (Table S1), the measured zinc concentrations in the samples exceed the limits set for Italy, Ontario, and Canada, particularly in Sample 2 (3465 mg/kg), which surpasses all the listed limits. Copper levels are generally within the acceptable range for most countries, although some samples exceed the Canadian limit. Lead concentrations tend to exceed the limits for Italy and Ontario, especially in Samples 2, 6, and 8, but remain within the limits for countries like Flanders. While cadmium limits are not provided in the first table, the measured cadmium levels are relatively low compared to the table.

The amounts of bioavailable metal forms extracted using 1 M ammonium nitrate in the examined soils were as follows: between <0.0050 and 0.03 mg/kg for Cd, <0.0050 mg/kg for Pb; 0.25 to 0.95 mg/kg for Cu, and 0.05 to 2.55 mg/kg for Zn (Table 2). Despite the high total concentrations of Zn in soils of several thousand mg/kg, the amounts of easily soluble Zn forms (released from soils in extractions with 1 M NH_4_NO_3_) remained low. In neutral soil (pH 6–7), which occurs in the study area, NH_4_NO_3_ was extracted only from 0.01% to 0.14% of the total zinc content (Table 2).

The use of this 0.43 M HNO_3_ extraction results in the release of 11 to 82% of total Zn, 30 to 83% of total Cu, 32 to 86% of total Pb, and from 35% to more than 90% of total Cd (Table 2). The highest concentrations of all trace elements extracted with 0.43 M HNO_3_ were found in soils 4, 5. The soil from sampling point 4 has the lowest organic C content of all garden soils. Both Cu and Pb are known for their high affinity binding to SOM.

### 3.2. The Ecotoxicity Indices

The mortality of test organisms after 6 days of incubation was low, not exceeding 30% in all samples (Table S2).

**Figure 2:**
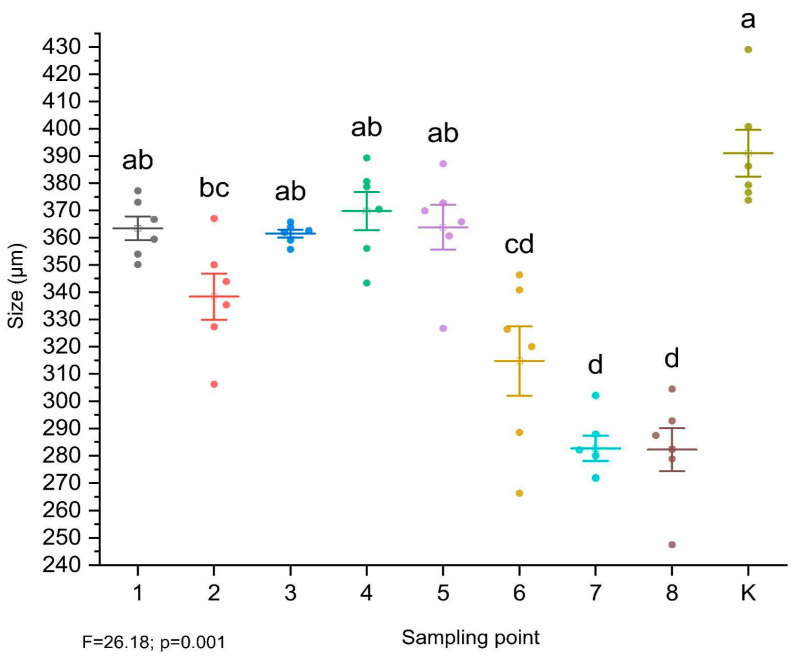
The length of the test organisms in μm after 6 days of incubation. Values were compared using ANOVA (*p* < 0.05). The significance of the differences between sampling points was evaluated using the post hoc Tukey test. Different lowercase letters on the graph indicate significant differences between treatments.

The growth of the test organisms was significantly inhibited at all sampling points in comparison to the reference soil (K) (F = 28.13, *p* = 0 < 0.001). The highest growth inhibition was observed for sampling points 7 and 8 and after that, 6 (Figure 2). More specifically, a significant reduction in organism growth was found in these sampling points compared to reference sediment (K) and control soil (1). Furthermore, no significant differences were found between the uncontaminated soil (1) and the soils from sampling points 3, 4, and 5.

Additionally, it should be noted that the highest levels of Pb contamination were identified in the soils from sampling points 7 and 8 (Table 1). The combined content of Pb, Cu, and Zn probably influenced the reduced development of ostracods in these areas. Furthermore, at sampling points 6 and 7, Zn concentrations exceeded the permissible standards, which may have contributed to the observed adverse effects on ostracods (Table 2).

The Pearson’s correlation analysis (Table 3) revealed that Pb is the most influential factor in growth inhibition, with strong correlations observed for both total forms and those available in NH_4_NO_3_ (r = 0.67 and 0.28, respectively). On the contrary, Cd and Zn exhibited significant negative correlations with growth inhibition, although they did not influence the negative effects on Ostracods. No significant correlation was found for Cu.

**Figure 3:**
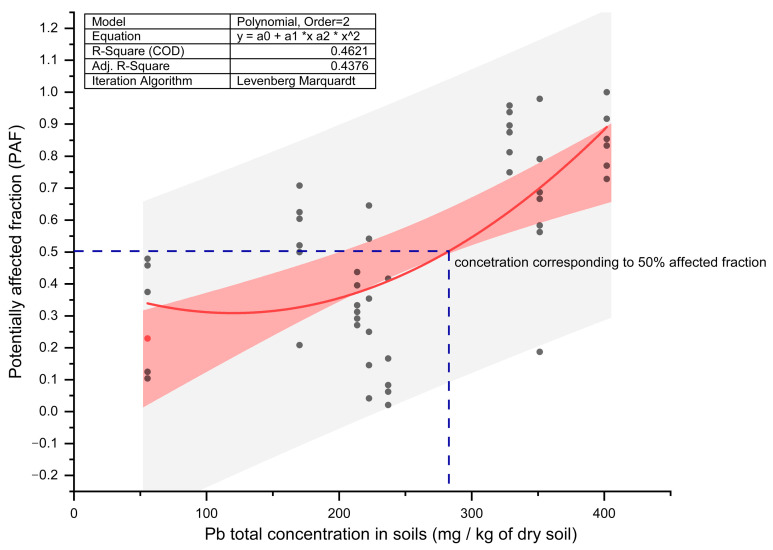
Species sensitivity distribution (SSD) of *Heterocypris incongruens*: relationship between the potentially affected fraction (PAF) of individuals (based on Growth Inhibition Response) and total Pb concentrations in Soil. Note: The pink band represents the confidence interval, the grey band represents the prediction interval, and the red curve represents the fitted polynomial model.

To assess the sensitivity of *Heterocypris incongruens* to heavy metal contamination, specifically to total lead (Pb) concentrations, in the soil, a species sensitivity distribution graph (SSD) was constructed (Figure 3). SSD highlights the non-linear relationship between Pb concentrations and the potentially affected fraction (PAF) of individuals, based on growth inhibition responses. As the Pb concentration increases, the response varies significantly, with a correlation coefficient (r^2^) of 0.46, indicating a moderate fit of the model. The graph also identifies the concentration of Pb corresponding to a 50% affected fraction of the species, which was determined to be 280 mg of Pb per kg of soil. This concentration marks a critical threshold where half of the test population is expected to experience growth inhibition due to Pb contamination.

## 4. Discussion

This investigation aimed to assess the environmental risks associated with trace element contamination in garden soils, using *Heterocypris incongruens* as a bioindicator. The findings provide valuable information on the complex interactions between soil contamination and ecological health, underscoring the importance of integrating ecotoxicological assessments into environmental monitoring.

### 4.1. Heavy Metal Contamination and Bioavailability

The presence of heavy metals, particularly zinc (Zn), cadmium (Cd), and copper (Cu), was found to exceed the allowed limits in urban garden soils and the high lead (Pb) content. Previous studies suggest that Pb contamination in these areas may arise from various anthropogenic sources, such as lead-containing paints, historical use of leaded fuels, and coal heating [60]. Waste incineration and metal-smelting activities are known contributors to Pb pollution [61].

Other investigations revealed that 16 of the 19 soil samples collected from gardens near the Zletovska River and a Pb-Zn mine exceeded Dutch environmental standards for heavy metals, emphasising the need for regulatory frameworks in regions lacking soil contamination guidelines. For example, the maximum concentration of Cd recorded was 4.03 mg/kg, while the levels of Pb were consistently high, indicating ongoing anthropogenic contamination in river sediments. Similarly, allotment gardens in Wroclaw, Poland, experienced contamination due to the proximity to metallurgical and automotive industries, illustrating a common industrial impact trend on soil quality [62].

The bioavailability of these trace elements is crucial to understanding their environmental risk. Bioavailability assessments typically rely on measuring labile trace element concentrations in the soil, which provides a more accurate reflection of potential toxicity than total concentration measurements alone [39]. This study aligns with the findings of [63], highlighting the significance of soluble forms of toxic elements in determining the environmental risk posed by soil pollution.

Chemical extractants, such as 0.01 M CaCl_2_ and 1 M NH_4_NO_3_, are effective in estimating the bioavailability of trace elements [63]. Despite the high total concentrations of Zn in the studied soils, only a small fraction was bioavailable, further confirming that the chemical forms, rather than the total amounts of metals, dictate environmental risk. Previous studies have shown that even elevated concentrations of metals do not necessarily correlate with increased leaching, plant uptake, or adverse effects on soil organisms [64].

The 0.43 M HNO_3_ extraction method has been shown to effectively assess the reactive fractions of trace metals [65]. This method proved useful in correlating the availability of Cd, Ni, and Zn with other soil attributes, providing a comprehensive view of trace metal behaviour in various soil types [66]. Other studies have also highlighted differences in the content of metals extracted from soils with 0.43 M HNO_3_, particularly with respect to organic carbon content [67].

### 4.2. Ecotoxicity Tests

The significant growth inhibition observed in *Heterocypris incongruens* at multiple sampling points highlights the effectiveness of using bioindicators to detect sublethal effects from trace metal contamination, often overlooked by standard chemical assessments. Lead was identified as the primary contaminant associated with growth inhibition, corroborated by correlation analyses. This finding underscores the ability of Pb to disrupt biological processes, even at low bioavailable concentrations, consistent with previous research that demonstrates its toxic effects on soil organisms [37,65]. The species-sensitivity distribution (SSD) analysis further supports this, showing a moderate correlation, which suggests that, while Pb concentration is a significant factor affecting the growth of *Heterocypris incongruens*, other environmental or biological factors, as well as the presence of other heavy metals, may also influence the species’ sensitivity. The observed non-linear trend is consistent with the behaviour of many ecotoxicological stressors, where lower concentrations may have minimal impact, but higher concentrations lead to more pronounced effects [68]. Identification of the 50% affected fraction (280 mg/kg of Pb) serves as a pivotal threshold for evaluating ecological risks, but this value should be viewed as a guide rather than a definitive risk cutoff.

Zinc and copper also contributed to growth inhibition; however, this relationship was not confirmed by the correlation analysis. Previous studies have demonstrated a link between high levels of bioavailable zinc and significant ecotoxicity, further supporting the potential role of these metals in affecting the health and growth [64,67]. Although mortality rates among ostracods were low across samples, observed growth inhibition emphasises the importance of considering both acute and sublethal effects in environmental risk assessments, as highlighted in previous studies [66,69].

The complexity of trace metal contamination in soils is further compounded by interactions between multiple metals, which can result in synergistic, antagonistic, or additive effects on organisms. In this investigation, the mechanisms of these interactions were not studied, but the presence of metal mixtures can alter the bioavailability of individual metals, affecting their toxicity and the overall ecological impact [70]. Understanding how metal speciation, soil properties, and biological effects interact is crucial for accurately assessing the risks posed by contaminated environments. Metal mixtures often lead to more unpredictable and potent toxic responses compared to individual metal exposures, highlighting the need for more comprehensive studies on combined metal toxicity [71]. Research into these interactions will improve environmental risk assessments, allowing for more reliable predictions of ecological consequences and more effective management strategies for contaminated sites [32].

## 5. Conclusions

Ostracods (*Heterocypris incongruens*) have proven to be reliable bioindicators for assessing heavy metal contamination in soils. When the total concentrations and bioavailable forms of metals are examined, it becomes possible to identify their specific effects on soil organisms. Lead (Pb) emerged as the most toxic metal in this study, significantly inhibiting the growth of the test organisms and highlighting its major role in soil ecotoxicity.

Localised contamination by heavy metals such as Zn, Cu, Pb, and Cd was identified in certain allotment gardens, posing potential risks to both soil ecosystems and human health. In addition to total metal concentrations, it is crucial to consider the bioavailability of metals and other factors that influence their mobility, including soil pH, organic matter content, and the origin of fertilisers used by gardeners, when assessing environmental risks.

The ongoing monitoring of these gardens is essential to assess the long-term ecological risks posed by heavy metal contamination and its broader impacts on both the environment and human health.

## Figures and Tables

**Table 2:** Contents of total and available forms of heavy metals in the examined soils (extraction in 1 M NH_4_NO_3_ and 0.43 M HNO_3_).

**Total Concentration of Selected Trace Elements (mg/kg, Soil Dry Mass)**				
**No. of Sampling Point**	**Zn**	**Cu**	**Pb**	**Cd**
1	222.00	45.00	56.00	1.00
2	3465.00	157.00	170.00	4.00
3	1739.00	322.00	214.00	5.00
4	1384.00	232.00	237.00	6.00
5	1290.00	219.00	222.00	6.00
6	1304.00	266.00	351.00	2.00
7	1098.00	216.00	329.00	2.00
8	1059.00	234.00	402.00	2.00
**Available Forms of Trace Metals Extracted in 0.43 M HNO_3_ (mg/kg, Soil Dry Mass)**				
**No. of Sampling Point**	**Zn**	**Cu**	**Pb**	**Cd**
1	-	-	-	-
2	394.40	77.30	119.90	2.90
3	1079.00	140.30	132.60	3.50
4	1090.00	186.20	206.70	6.10
5	1060.00	182.20	189.60	6.30
6	486.60	81.00	126.80	0.70
7	493.40	85.30	158.40	0.70
8	613.20	96.40	129.10	0.80
**Available Forms of Trace Metals Extracted in 1 M NH_4_NO_3_ (mg/kg, Soil Dry Mass)**				
**No. of Sampling Point**	**Zn**	**Cu**	**Pb**	**Cd**
1	-	-	-	-
2	0.55	0.25	<0.0050	<0.0050
3	2.55	0.50	<0.0050	<0.0050
4	0.85	0.68	<0.0050	0.03
5	0.65	0.95	<0.0050	0.03
6	0.20	0.28	<0.0050	<0.0050
7	0.18	0.30	<0.0050	<0.0050
8	0.05	0.43	<0.0050	<0.0050

Note: Heavy metal concentration values that exceed the permitted limits established in Polish legislation [54] are highlighted in grey cells; permissible limits for Zn: 1000 (mg/kg), Cu: 300 (mg/kg), P: 500 (mg/kg), Cd: 5 (mg/kg) in clay soils with a neutral reaction used to grow plants and vegetables. Note: In July 2014, the Polish Law on Environmental Protection and the Law on Prevention of Environmental Damage and its remediation introduced new provisions, changing the approach to assessing the state of soil contamination and the principles of remediation, incorporating environmental risk assessment.

**Table 3:** Pearson correlation between growth inhibition and total, as well as available, forms of heavy metals in examined soils (extraction in 1 M NH_4_NO_3_ and HNO_3_).

**Zn**		
	**Pearson Corr.**	***p*-Value**
Total	−0.0552	0.7092
Available in NH_4_NO_3_	−0.4360	0.0020
Available in HNO_3_	−0.2899	0.0456
**Cu**		
	**Pearson Corr.**	***p*-Value**
Total	0.1447	0.3266
Available in NH_4_NO_3_	−0.2712	0.0623
Available in HNO_3_	−0.2772	0.0565
**Pb**		
	**Pearson Corr.**	***p*-Value**
Total	0.6715	<0.0001
Available in NH_4_NO_3_	0.2858	0.0489
Available in HNO_3_	0.0356	0.8103
**Cd**		
	**Pearson Corr.**	***p*-Value**
Total	−0.5406	<0.0001
Available in NH_4_NO_3_	−0.2782	0.0555
Available in HNO_3_	−0.5753	<0.0001

## Data Availability

Data will be made available on request.

## Supplementary Materials

The following supporting information can be downloaded at: https://www.mdpi.com/article/10.3390/toxics12120873/s1, Table S1: Limits for Cu, Zn (mg/kg, dry soil) for Agricultural Land Uses in Various Countries [56,57,58,59]; Table S2: The mortality of the test organisms after 6 days of incubation (%).
