# Supplementary material for: Assessing Environmental Risks of Local Contamination of Garden Urban Soils with Heavy Metals Using Ecotoxicological Tests
Source: Toxics. 2024 Nov 30;12(12):873. doi: 10.3390/toxics12120873 (PMC11679028; doi:10.3390/toxics12120873)
Supplement: Supplementary file 1 [file toxics-12-00873-s001.zip › toxics-3318811-supplementary.pdf]

toxics MDPI

Supplementary Materials: Assessing Environmental Risks of Local Contamination of Garden Urban Soils with Heavy Metals Using Ecotoxicological Tests

Dariusz Gruszka, Iwona Gruss and Katarzyna Szopka

Table S1. Limits for Cu, Zn (mg/kg, dry soil) for Agricultural Land Uses in Various Countries.

Country	Cu	Zn	Pb	Cu	References
Italy (SV)	120	150	100	2	[56]
Czech R. (AV)	600	2500		20	[56]
Flanders (IV)	400	1000	700		[57]
Ontario-Ca (SS)	140	340	120	1,2	[58]
Canada (SQG)	63	250			[59]

Note: Abbreviations: **SV** = Screening Value; **IV** = Intervention Value; **TV** = Trigger Value; **AV** = Action Value; **SS** = Stratified Site Condition Standards in a Non-potable Groundwater Conditions; **SQG** = Soil Quality Guidelines.

Table S2. The mortality of the test organisms after 6 days of incubation (%).

Replication	1	2	3	4	5	6	7	8	K
1	0	0	0	0	10	0	0	0	0
2	0	0	0	0	10	0	10	10	0
3	0	0	0	0	0	0	0	10	0
4	0	0	0	0	0	0	0	10	0
5	0	0	0	0	0	0	10	0	0
6	0	0	0	0	0	0	70	0	0
Mean mortality	0	0	0	0	3.33	0	15	5	0
